# On the Use of Sulphate of Quinia

**Published:** 1848-12

**Authors:** R. L. Scruggs

**Affiliations:** Germantown, Tennessee


*On the use of Sulphate of Quinia.* By R. L. SCRUGGS, M. D., of Germantown, Tennessee. Extract of a letter addressed to Mr. *W. L. Adkins*, Student of Medicine in Jefferson Medical College.

I promised to give you my views upon sulphate of quinia; the various pathological conditions in which it might be used advantageously; and the circumstances under which it ought to be administered.

Perhaps the discovery of no article in the materia medica, has conferred such signal benefits upon mankind; and although this opinion is almost universally entertained by the profession, yet a greater diversity of opinion, probably does not exist, in regard to any other article, as to the precise circumstances under which it should be administered; particularly in the treatment of the bilious remittent fevers of our country.

I hold it to be a most important fact connected with the use of this valuable medicine, that it should never be given upon a rising fever; i. e. until the fever has attained its climax, or has been arrested by the use of the lancet or other remedial agents, and has a downward tendency. Then, although the pulse may have mounted up to one hundred and forty beats to the minute, and have declined only ten beats, quinine may be given in large and repeated doses, combined with ipecac., not only with safety, but with the most decidedly beneficial results. This particular fact connected with the use of the article, is the one that I desire to be especially noticed, as I have not seen the idea advanced by any writer upon the subject. Prof. Wood, in his recent work on Practice, comes nearer to it than any other writer that I have had access to; and he is undoubtedly correct when he says that "the doses must be regulated by the length of the remission. If this be short, they must be very large; and if of a few hours duration only, the whole quantity must be taken in two or three doses."

I repeat, if you give quinine when the pulse is at one hundred and thirty beats to the minute, and falling, (premising of course the necessary depletions,) and give it in sufficient quantity, say five grains, with a little ipecac. every hour, that you will, in a very large majority of cases, find that the pulse will fall rapidly under its influence, until by the time the fifth portion is exhibited, your patient is altogether free from fever. Whereas, if you give it when the pulse is at eighty beats to the minute, and rising, you will invariably increase the action of the heart in force and frequency, and if pushed to any considerable extent, cause the death of your patient. It is also well worthy of remembrance, that quinine given at an improper time, is not only calculated to excite fever, and do an immense amount of injury in that way, but oft repeated observations have confirmed me in the opinion, long since entertained, that it materially lessens the capability of that article to impress the system favourably afterwards; i. e. after you have succeeded in effecting a remission or intermission of the fever; thus depriving us of the use, to a certain extent, of the most valuable agent known to medicine in combatting this dangerous malady.

I made these observations several years ago, in the commencement of my practice in this country, since which time I have had ample opportunities of testing their value; and I have the satisfaction of knowing that the practice predicated upon these opinions, has enabled me to contend successfully, and with comparative ease, with the most malignant forms of the bilious remittent and congestive fevers of this climate.

Writers upon this subject frequently urge the necessity of giving large doses of quinine in the fever, but fail to notice, as I believe, the most important circumstances connected with it, and thus are not unfrequently disappointed in their hopes and expectations, but ignorant of the causes that led to the failure. Thus we find one physician lauding to the skies the plan of giving quinine in the fever, and another equally entitled to our respect, depreciating it in the most unmeasured terms. These discrepancies in the statements and opinions of medical men, with equal opportunities of judging correctly, have caused, in some, a too timid use of the article, while others have been encouraged to administer it with a liberal hand, succeeding sometimes, and sometimes failing, which failure or success must, if I am right in my views, be altogether accidental. With these general remarks upon the use of quinine, I will endeavour to comply with your request by giving you my treatment of bilious remittent fever, as most commonly met with in this climate; as well as the treatment of the congestive form of the same disease. Congestion, you will bear in mind, is only a condition, and does not constitute a distinct disease of itself, but may exist in either of the great splanchnic cavities, in either type of ague, and in several kinds of fever; a circumstance that must always greatly influence your treatment of the disease under consideration, or any other disease with which it may be found to exist. Should your patient be a robust person, with a full, bounding pulse, dry, hot skin, &c., you should not hesitate to use the lancet freely,—placing the patient in the erect or semi-erect position, and letting the blood flow until a decided effect is produced. Then, if the stomach is irritable or inflamed, or if there is, as will often be found in these cases, great uneasiness in the epigastric and right and left hypochondriac regions, with difficult respiration, and a feeling as of a heavy weight pressing upon them, indicating congestion of the viscera of these parts,—I would scarify and cup freely, or until these uneasy sensations are somewhat or altogether relieved; if, however, the cups fail to procure the desired relief, I would then apply warm fomentations, after which frictions of some stimulating ointment, and lastly, a pepper mush poultice, to remain on for several hours; which may be renewed, if required. While this is being attended to, see that the extremities are kept warm. A stimulating foot bath, with cups to the temples, or behind the ears, are the best means that can be used to relieve the pain of the head, so constantly attendant upon this disease. You will then give calomel, nitrate of potash and ipecac. every two hours, (about two grains of calomel at a dose,) alternating this with spirits. nitr. dulc. and vin. ipecac., so that the powders or drops shall be given every hour. Four of these powders should be given, unless the bowels are moved before the last portion is taken. If they should fail to move the bowels in six or eight hours after the last portion is administered, then give a teaspoonful of castor oil, with fifteen or twenty drops spts. turpentine, mixed with a little white of egg or elm water, and repeat every two hours until the bowels are moved. The nitrous powder is often objected to on account of its irritating effects upon the stomach, but if you will cup over the stomach, and apply a pepper or mustard poultice *first*, and then give the powder, with a little *dry* sugar, instructing the patient to swallow it *quickly*, with water or tea, before the nitrate has time to dissolve in the mouth, you will rarely, if ever, fail in accomplishing your object. Should the stomach, however, reject the powders in spite of all your efforts, the nitrate of potassa must of course be omitted. But the good effects of this combination are so striking in quickly reducing the action of the heart, and producing moisture upon the skin, that I would always make the effort to have it retained upon the stomach.

When with these means you have succeeded in reducing the pulse to its natural standard, or failing in this, you succeed in reducing the fever to a considerable degree, you may commence the use of quinine in five grain doses every hour, watching its effects carefully the while, and stopping its use as soon as you perceive the slightest tendency to a rise of fever. If the quinine acts well; in other words, if you have commenced its use at the proper time, not mistaking a tendency to decline, you will find the fever gradually abate under its influence, the skin become relaxed, and the pulse reduced to its natural standard, and even sometimes far below it. A singular case of this latter effect of quinine I will now briefly relate.

Visited Tom, a negro slave, aged about thirty years, in the spring of 1846; found him with pneumonia of the lower half of the right lung—treated it in the usual way, and succeeded in a few days in removing the inflammation; but to my surprise, after all evidence of pneumonia had entirely disappeared, the pulse mounted up to one hundred and thirty beats in the minute, and seemed to remain at that, night and day. But by a careful examination and inquiry, I made out fever of the double tertian type, and prescribed quinine, grs. v.; ipecac, gr. s., to be given every hour for five times, commencing as soon as the pulse should fall below one hundred and thirty—and to repeat this the next day—commencing in time to impress the system with the article before the hour for the fever to rise. At my next visit, I found the patient free from fever, and doing well in every respect. The owner of the slave informed me that he had only given three portions of quinine the day before, and that his reason for not carrying out my directions fully, was that the pulse fell so rapidly under its use, that he was fearful the heart would cease to beat altogether; it having fallen in four hours from one hundred and thirty to forty-four beats in the minute. After this, a few portions of quinine (properly timed) every day, for three or four days, lessening the quantity each day, sufficed to effect a cure. This is rather a singular case, for a philosophic explanation of which, I must of necessity refer you to your professors, as I am unable to give it myself.

You will frequently be called to patients, particularly among the poorer classes of this country, where the disease has been permitted to run on for several days without treatment. They do not send for you until the case becomes alarming. You find the patient insensible—comatose ; head and body hot, flushed face and dry hot skin ; cold extremities, pulse feeble, and so fast that you cannot count it. You inquire into the history of the case, and are told that the patient was taken with a slight chill several days previously, and that the fever has continued ever since without intermission or remission. This you will conclude is an incorrect statement, the result of ignorance or lack of close observation. That there has been exacerbations and remissions you may be sure of. The first thing to be done in a case of this kind, is to immerse the patient's feet and legs in a tub of hot water, made very stimulating with pepper, mustard or salt; where they should remain until the head symptoms are relieved.

Then cup the temples and behind the ears, after which pour cold water upon the head until the patient is sensible, always recollecting to desist in your cold applications as soon as the patient complains of its producing cold or other unpleasant sensations. When this is done, you will ascertain if there be any distress about the stomach, liver or spleen, and if so, cup over those organs, and apply mustard or pepper poultices. Immersing the whole body in a warm or tepid salt bath, protecting the body at the same time by wrapping in a blanket, while you pour the cold water on the head, will often be requisite, and answer an admirable purpose: or, keeping the feet in the hot bath, sponge the body with warm soap suds, and pour the cold water on the head. If by the application of these means, you succeed quickly in reducing the fever and relieving the head, you may commence with the quinine and ipecac. and give it every hour for five or six times, to each dose of which, if no medicine has been previously given, add two or three grs. calomel: but if you fail in relieving the head, and particularly if the patient is young—under twelve years old, and has had convulsions, is breathing stertorously and apparently sleeping, with one or both eyes half closed, you must reject quinine altogether, and rely upon the blood letting, general and topical, and the assiduous application of all the other means just pointed out. I have had a considerable number of these cases this season; and I have used but little quinine in the treatment of any of them. I rely upon venesection, the general warm bath, cups to the temples, and last, though not least, cold water to the head. These are the external agents; internally I give *calomel and ipecac.* every two, three, or four hours, according to circumstances; the object being in the first place to move the bowels freely, and afterwards to keep up a constant but gentle action upon them. The calomel and ipecac. fulfil the indications here more perfectly than any other articles can do. They empty the bowels, promote the secretions of the liver and all the other glands along the course of the alimentary track; relieve the brain by their sedative influence upon the action of the heart, by direct depletion, by promoting the secretions, and enabling the absorbents to take up the affused fluids. After the more acute stage is passed, you must not neglect the use of blisters. They should be applied to the upper and lower extremities, and nape of the neck, and should be kept running until the patient is altogether free from danger. Instead of applying the blister to the ankles, as is usual, I would advise its application to the upper and inner part of the calf of the leg; so that you might, if necessary, have the benefit of the foot bath without interfering with the blister.* These violent cases of inflammation or congestion of the brain in children, so common in this country, constitute an important class, the treatment of which should be well understood by the physician. He should be able to recognize them at once, understand distinctly the indications to be fulfilled, and act promptly and energetically. They differ from those cases that supervene upon inflammation of the stomach and bowels, and are said to be reflected upon the brain; the prognosis is generally more favorable, the treatment required more energetic, and the management of them less delicate. They appear not to be caused by malaria, or any other specific agent; although they may assume, in their course, a periodic character, and require quinine in the treatment, but the phenomena presented are those of simple inflammation or congestion of that organ, which generally yields to an energetic antiphlogistic treatment and revulsives. The immediate cause of the disease is probably irritation from worms in the bowels, indigestible food, or exposure to the sun. Again, it differs widely from another disease of the brain and spinal marrow, which, fortunately, is not so common in this country; the pathology of which is indicated by its name, to wit: cerebro-spinal-meningitis, a history of which fearful malady, as it has occurred here this year, I must reserve for another letter.

I will now attempt to give you some idea of our treatment of the so-called congestive fevers of our country, in the cold stage. The after treatment, you will be able to learn much better than I can teach, in the schools, from the books and by your own observation. Indeed, most of the writers describe this part of the treatment with sufficient accuracy, but I think they fail to point out with clearness, the *extent* necessary to carry the means that are to be used, and it is to this that I would call your particular attention. You will bear in mind that there is great

* The same remarks will apply to the use of sinapisms in the first stage.

danger of death in the first or cold stage, in the malignant cases; re-action never taking place unless nature is assisted by art. So insidious are these attacks sometimes, that the patient is not aware of it himself for some time after it commences. The legs and arms gradually become cold, the coldness commencing at the free extremity of the limb, and gradually extending up to the body. He is at last reminded of his condition by a sense of great weight or oppression about the epigastrium, which continues to deepen until he is utterly insensible to all surrounding objects. Sometimes I have seen patients with all the evidences of deep congestion, without the intellectual faculties being at all impaired—they complain of no particular pain, but of an indefinable sense of misery. I will not stop to theorize upon the probable cause of these malignant diseases, for of this you will find more than enough already written. It is doubtless the introduction of some subtle poison into the system, causing deep lesion of the nervous centers, affecting thereby calorification, and preventing the heart from sending the blood to the extreme capillaries. Differences in age, sex, constitution, idiosyncrasy, &c., will cause differences in the phenomena presented in different cases, but the indications of treatment are nearly the same in all. They are to warm the extremities, equalize the circulation, relieve the congestions and inflammations, allay nervous irritability, and put a stop to the periodic tendency of the disease. To accomplish which, external heat, cupping over the epigastric and right and left hypochondriac regions and along the spine, warm fomentations, the application of sinapisms to the extremities, spine, chest and abdomen, promoting the secretions, and to fill the two last indications, to introduce a sufficient quantity of the antiperiodic agent and morphine: all these may be brought into requisition, and to the *extent justified by the urgency of the symptoms.* When called to treat one of these cases, I direct eight or ten gallons of water to be heated, ears of boiled corn, bricks heated; and a pint of spirits of turpentine, with an equal quantity of vinegar, to be put into a vessel and made boiling hot, into which put half pound of mustard and the same quantity of cayenne pepper. With this strips of flannel are to be saturated, and the extremities enveloped from the fingers and toes to the trunk, and then the heated mixture to be poured upon them. A small blanket torn into strips of the proper width answer the best purpose for encircling the limbs. The hot bricks should then be put about the feet and legs, and the hot corn about the body. Previously, however, to doing this, the patient should be cupped over the liver and stomach, and the warm fomentations used; also, cupped along the spine, and the sinapisms applied along the spine from the occiput to the sacrum, and over the lower part of the chest and abdomen.

The most important inquiry now is, at what time, and in what quantity, shall quinine be given? This will depend upon circumstances. If we have reason to believe that it is a congestive ague of the quotidian type, we need be in no great hurry about administering quinine; but as we cannot always arrive at a correct conclusion upon this point, it will be safest to commence giving it as soon as there is a perfect intermission. But if it be congestive remittent fever, I hold that a perfect re-action should *first* be established, and then at the first appearance of a decline of the pulse, the quinine should be commenced. About five grains every hour for five or six times is the quantity that I usually give; and eight grains, I think, is the largest amount that I have ever given at a dose in these cases.

In conclusion, I will say that it would be extremely difficult to enumerate all the cases of disease, and conditions of body that may be benefitted by the use of quinine. But, regarding it as a pure tonic in small doses, and as *the* great anti-periodic agent, I should say that its use was indicated in nearly all cases requiring a tonic treatment, alternating or combining it with other tonics; and in all cases of disease that commence with, or assume during their course, the periodic character. Of these latter, you will find a vast many in the South; for, in the language of some of the ancient writers, intermittent fever, at times, seems to have the power of forcing all other diseases to put on its livery.

